# Compound heterozygous variants including a novel copy number variation in a child with atypical ataxia-telangiectasia: a case report

**DOI:** 10.1186/s12920-021-01053-3

**Published:** 2021-08-17

**Authors:** Hoo Young Lee, Dae-Hyun Jang, Jae-Won Kim, Dong-Woo Lee, Ja-Hyun Jang, Joungsu Joo

**Affiliations:** 1 TBI Rehabilitation Center, National Traffic Injury Rehabilitation Hospital, Gyeonggi-do, Republic of Korea; 2 Department of Rehabilitation Medicine, Seoul National University Hospital, Seoul, Republic of Korea; 3 Department of Medicine, Yonsei University College of Medicine, Seoul, Republic of Korea; 4 National Traffic Injury Rehabilitation Research Institute, National Traffic Injury Rehabilitation Hospital, Yangpyeong, Korea; 5 Department of Rehabilitation Medicine, Incheon St. Mary’s Hospital, College of Medicine, The Catholic University of Korea, 56, Dongsu-ro, Bupyeong-gu, Incheon, 21431 Republic of Korea; 6 Department of Laboratory Medicine and Genetics, Samsung Medical Center, Seoul, Korea; 7 EONE-DIAGNOMICS Genome Center, Incheon, Republic of Korea

**Keywords:** Case report, Ataxia telangiectasia, DNA copy number variation, Pathologic variants

## Abstract

**Background:**

Ataxia-telangiectasia is a rare autosomal recessive, neurodegenerative disorder caused by alterations in the *ATM* gene. The majority of *ATM* pathogenic variants are frameshift or nonsense variants which are predicted to truncate the whole ATM protein. Herein, we report on an ataxia telangiectasia child with atypical phenotype who was identified as compound heterozygous for two *ATM* variants involving a previously described pathogenic single nucleotide variation (SNV) and a novel copy number variation (CNV).

**Case presentation:**

A 6-year-old boy presented with delayed development and oculomotor apraxia. Brain magnetic resonance imaging showed interval development of mild atrophy in the cerebellum. Serum alpha fetoprotein level was in normal range. Next-generation sequencing and single-nucleotide polymorphism array tests were performed. Next-generation sequencing revealed a heterozygous nonsense pathogenic variant in *ATM*, c.742C > T (p.Arg248Ter) inherited from the father. Single-nucleotide polymorphism array revealed a compound heterozygous CNV, arr[GRCh37] 11q22.3(10851766–108183226) × 1, 31460 bp (exons 24–40 deletion of *ATM*) inherited from the mother, which was validated by reverse transcription-polymerase chain reaction analysis (RT-PCR). We demonstrated that this variant (NM_000051.4:c.3403_6006del) generated a product of in-frame deletion of exon 24–40 of *ATM* (p.Ser1135_Gln2002del).

**Conclusions:**

The compound heterozygosity for *ATM* variants involving a previously described pathogenic SNV and a novel CNV may be associated with the atypical clinical manifestations. This clinical report extends the genetic and phenotypic spectrum of *ATM* pathogenic variants in atypical ataxia-telangiectasia, thus making implementation of advanced analysis beyond the routine next-generation sequencing an important consideration in diagnosis and rehabilitation services for children with ataxia-telangiectasia.

**Supplementary Information:**

The online version contains supplementary material available at 10.1186/s12920-021-01053-3.

## Background

Ataxia-telangiectasia (A-T) is a rare autosomal, recessive disorder characterized by complex, degenerative neurological dysfunctions related to multisystem abnormalities [1]. Classical neurological signs of A-T include progressive cerebellar ataxia, oculomotor apraxia, abnormal limb movement, and cognitive impairment. Multisystem involvement, including immunocompromised states, progressive respiratory failure, radiosensitivity, oculocutaneous telangiectasia, and an increased risk of malignancy with elevated serum alpha fetoprotein (AFP) levels, leads to complications [2–5]. Studies have reported a wide range of phenotypic manifestations involving the classical phenotype as well as atypical clinical presentations that are not usually seen with A-T [6–11]. Therefore, Teive et al. recommended that A-T be renamed “ataxia telangiectasia mutated” (ATM) syndrome [12, 13].

A-T is caused by pathogenic variants of *ATM* which belongs to the phosphatidylinositol 3-kinase–related protein kinase (PIKK) family. It codes a Ser/Thr kinase that is involved in DNA repair and that phosphorylates substrates involved in cell signaling to regulate the cell cycle, repair double-stranded DNA breaks, react to oxidative stress, and control transcription [14]. The demonstration of pathogenic variant proves the clinical diagnosis of A-T. The majority of *ATM* pathogenic variants are single-nucleotide variant (SNV) alterations such as frameshift or nonsense pathogenic variants, which are predicted to truncate the whole ATM protein [15]. Other SNV pathogenic variants of *ATM* include missense variants, and splicing. According to data from databases such as the Human Gene Mutation Database (http://www.hgmd.cf.ac.uk/ac/gene.php?gene=ATM), the copy number variation (CNV) in *ATM* is detected in about 1 ~ 10% of A-T patients. Previous reports estimated that the large genomics alterations in *ATM* are detected in 1 ~ 12% of AT patients [16–18]. However, there is little information available for the co-occurrence of SNV and CNV as well as its identified role or phenotype burden in A-T patients. Herein, we report an A-T child with atypical phenotype who was identified as compound heterozygous for one *ATM* pathogenic variant and a novel CNV.

## Case presentation

A 6-year-old male patient was transferred to our clinic for proper diagnosis of ataxia, delayed cognitive and speech-language development, and oculomotor apraxia. The patient was the fourth son of nonconsanguineous healthy Korean parents, with an unremarkable family history, including cancer. He was born at 40 weeks’ gestation, and there were no other pre- or postpartum events. His birth weight was 4.08 kg. He started to stand by himself at 10 months of age, and at 12 months of age, he started to take two to three steps but immediately fell down. At our clinic, he could walk with hand support, but because of the ataxia in his trunk and limbs, he could not walk independently and fell down within five steps. He could climb a few stairs without holding the railing, but he had to hold the railing as he came down the stairs. He was able to jump, but the landing was unstable. He also showed some growth delay: his weight was 20.4 kg (5th percentile) and height 110 cm (< 3rd percentile). Physical examination revealed ocular telangiectasia; telangiectasia on his cheeks; and pigmented nevi along his cheeks, back, and arms without nodes or hepatosplenomegaly.

**Fig. 1:**
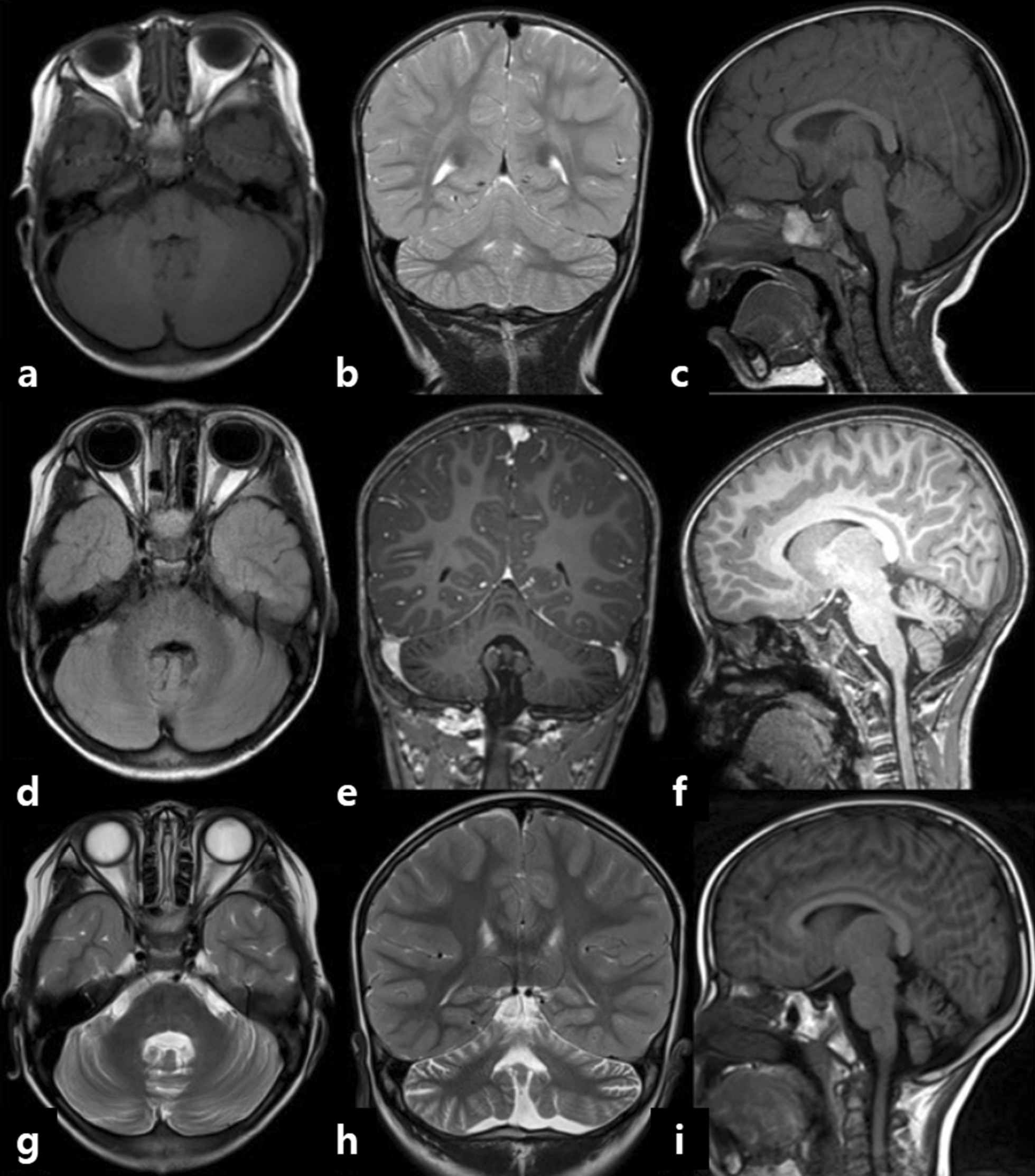
Brain MRI of the patient at 1 year (**a**–**c**), 5 years (**d**–**f**), and 7 years (**g**–**i**). Interval development of mild atrophy in the cerebellar vermis and hemispheres and subtle increased FLAIR signal intensity were noted. *MRI* magnetic resonance imaging, *FLAIR* fluid-attenuated inversion recovery

Neurological examination of the patient showed neurological dysfunction. The cranial nerve function test revealed nystagmus in both eyes, and the cerebellar function test showed an intentional tremor in distal extremities, with positive signs on the finger-to-nose and heel-to-shin tests. The patient could perform neither the Romberg test nor tandem gait because of ataxia. Deep tendon reflexes were decreased, and no upper motor neuron signs were detected. According to the Denver Developmental Screening Test 2nd edition, the age equivalents for the patient’s personal–social, fine motor and adaptive, language, and gross motor skills were 49, 60, 66, and 41 months, respectively. The percentage of the total score of Gross Motor Function Measure-88 was 88.56%. His Receptive and Expressive Vocabulary Test results were 20th percentile and < 10th percentile, respectively, and his Assessment of Phonology and Articulation for Children score was < 1st percentile. The Korean–Wechsler Intelligence Scale for Children 4th edition revealed a full-scale intelligence quotient of 0.4th percentile, a Verbal Comprehension Index of 1.7th percentile, a Perceptual Reasoning Index of 2.9th percentile, a Working Memory Index of 1.0th percentile, and a Processing Speed Index < 0.1th percentile. Brain magnetic resonance imaging (MRI) examination at 1, 5, and 7 years showed interval development of mild atrophy in the cerebellar vermis and hemispheres, with subtle increased fluid-attenuated inversion recovery signal intensity (Fig. 1). Immunology test results were borderline values and AFP levels were normal. Nerve conduction studies and electromyography showed distal symmetric sensory peripheral polyneuropathy of the upper and lower extremities. We proposed a genetic test to check for the possibility of congenital peripheral polyneuropathy manifesting ataxia such as spinocerebellar ataxia, Friedreich ataxia, and A-T [19].

**Fig. 2:**
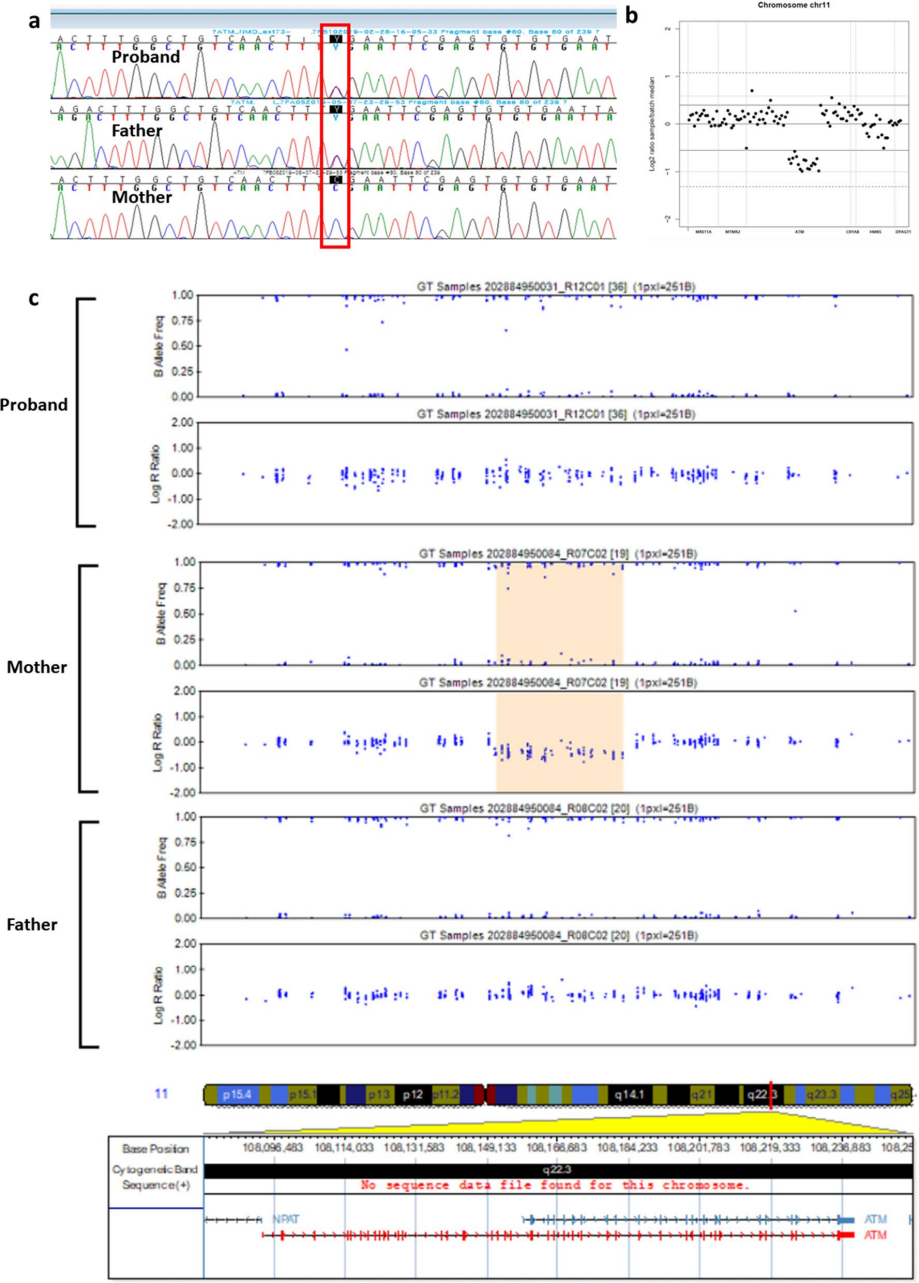
**a** Chromatograms of *ATM* sequence in the proband (top), the patient’s father (middle), and the patient’s mother (bottom) showing an SNV of c.742C > T (p.Arg248Ter) from the father. **b** Analysis of the next-generation sequencing data using VisCap c. SNP array analysis of the chromosome from the proband (top), the patient’s mother (middle), and the patient’s father (bottom) showing a novel CNV by the deletion of exons 24–40 from the mother. *SNV* single-nucleotide variation, *SNP* single-nucleotide polymorphism, *CNV* copy number variation

Next-generation sequencing (NGS) was performed by a targeted gene-sequencing panel that analyzed 410 ataxia-associated genes and other genetic neuromuscular diseases as described in the previous report [20]. Genomic DNA was extracted from the patient’s peripheral blood. Library preparation and target enrichment were conducted by hybridization capture. Custom oligo design and synthesis were performed by Agilent Technologies (Santa Clara, CA, USA). Massive parallel sequencing was performed with 2 × 150 bp in the paired-end mode of the NextSeq platform (Illumina, San Diego, CA, USA). NGS revealed a heterozygous nonsense variant in *ATM* (MN_000051.3), c.742C > T (p.Arg248Ter), which was related to A-T and was previously reported as pathogenic [21]. VisCap was used to detect CNVs from NGS data, based on the read depth approach, and one CNV of *ATM* was identified [22]. To validate the CNV, we performed a single-nucleotide polymorphism (SNP) Infinium Global Screening Array-24 + v2.0 array (Illumina, San Diego, CA, USA). A compound heterozygous CNV, arr[GRCh37] 11q22.3(108151766–108183226) × 1, 31460 bp (exons 24–40 deletion of *ATM*) was revealed (Additional files 1, 2). This CNV has not been reported in control databases. The nonsense variant was confirmed by Sanger sequencing, and the patient’s parents were diagnosed as heterozygous carriers for the trans variation using a NGS and a SNP array (Fig. 2). To investigate the potential pathogenicity of the aberrant deletion, total RNA was isolated from peripheral blood from the patient and his mother and used the RT-PCR method with RiboPure blood kit (Thermo Fisher Scientific) with informed consent. First-strand cDNA was generated with a SuperScript IV VILO kit (Invitrogen) according to the manufacturer's recommendations and was used as a template for PCR. The primer sequences used for RT-PCR were as follows: *ATM*_RT-F: 5'-atg ttg gct gca gag tca atc-3', *ATM*_RT-R: 5'-ttc tgc aag gcc tga atg at-3'. The PCR products were directly sequenced for both strands using the above primers. Sequencing analysis of this RT-PCR product disclosed a 2,289 base pair loss at the cDNA level. We demonstrated that this variant (NM_000051.4:c.3403_6006del) generated a product of in-frame deletion of exon 24–40 of *ATM* (p.Ser1135_Gln2002del) (Additional file 3).

**Fig. 3:**
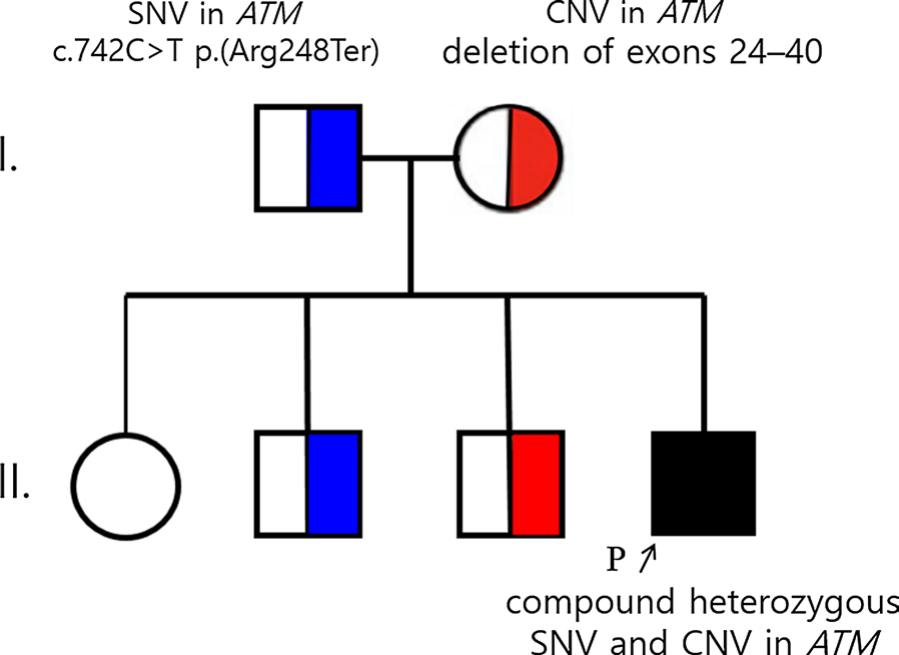
Family pedigree diagnosed with the compound, heterozygous *ATM* pathogenic variant. The black filled‐in pedigree member is the patient (c.742C > T (p.Arg248Ter) and deletion of exons 24–40), the blue half‐filled pedigree member indicates the heterozygous carrier, c.742C > T (p.Arg248Ter), whereas the red half‐filled pedigree member is the heterozygous carrier with a novel CNV, deletion of exons 24–40. *CNV* copy number variation

On the basis of our findings, the patient was finally diagnosed with A-T related to compound heterozygosity for an *ATM* pathogenic SNV and a new CNV. The deletion of exons 24–40 of *ATM* was inherited from the mother and the pathogenic SNV c.742C > T (p.Arg248Ter) from the father. Some of the patient’s siblings were confirmed as carriers (Fig. 3).

Inpatient rehabilitation service was provided for 3 months, which was switched to outpatient rehabilitation, continued for more than 2 years. Rehabilitative interventions included physical therapy to promote activity to preserve strength and endurance, occupational therapy to teach compensatory strategies to decrease effects of ataxia and tremor, cognitive therapy, and speech therapy to guide compensatory methods to relieve dysarthria. The patient continued the rehabilitation treatment without absenteeism, hence, adherence to the rehabilitation service was favorable. Neurological examination, Gross Motor Function Measure, Pediatric Balance Scale, immunological tests, and an AFP test were repeated after 2 years (Table 1). After two years, when the patient turned 8 years old, the percentage of the total score of Gross Motor Function Measure-88 declined to 64.02%. Immunology test results did not show significant changes overall, and the AFP level increased.

**Table 1:** Two-year follow-up of clinical and laboratory data of the patient

Assessment	At diagnosis	2 years follow-up
Age (year)	6	8
*GMFM-88 Dimension Subscore (Percentile, %)*		
A. Lying and rolling	50 (98)	51 (100)
B. Sitting	59 (98.3)	57 (95)
C. Crawling and kneeling	41 (97.60)	35 (83.33)
D. Standing	31 (79.50)	17 (43.59)
E. Walking, running, and jumping	50 (69.40)	9 (12.5)
GMFM-88 Dimension Total percentile (%)	88.56	64.02
Pediatric Balance Scale (point)	23	5
Total IgE (mg/dl)	< 0.0	< 1.5
IgG (mg/dl)	922.6	1033.7
IgM (mg/dl)	469.0	538.0
IgA (mg/dl)	12.4	29.6
CD3 (T cell) (%)	54.45	50.15
CD4 (T helper) (%)	23.85	21.35
CD8 (T suppressor) (%)	22.32	16.92
CD19 (B cell) (%)	9.14	11.04
NK cell (%)	33.51	33.34
AFP (ng/mL)	1.41	336.03

*GMFM* Gross Motor Function Measure, *NK cell* Natural killer cell, *AFP* Alpha fetoprotein

This case report was carried out in accordance with the recommendations of the institutional research review board of the Catholic University of Korea, Incheon St. Mary’s Hospital. The parents of the patient gave written informed consent for the publication of the case with anonymity in accordance with the Declaration of Helsinki. All variants included in this article have been submitted to the Clinvar.

## Discussion and conclusions

A-T or ATM syndrome shows clinical heterogeneity, from severe, early-onset classical forms related to the total loss of the ATM protein to delayed and attenuated or absent neurological progression or an adult-onset disorder that manifests atypical neurological abnormalities and fewer systemic symptoms [23–27]. For instance, gait instability may develop slowly and naturally becomes evident after 5 years of age [28]. In addition, brain MRI may be normal in the initial stages of the disease [29].

It has previously been suggested that the phenotypic variability in A-T can be explained by differences in genotype. Pathogenic variants leading to even small amounts of active residual ATM protein can rescue the patient from classic A-T [30]. With the development of molecular analysis, research on the relationship between the degree of phenotypes and residual ATM kinase activity has increased. Atypical phenotypes are related to the presence of residual ATM kinase activity, which is believed to be the key to carcinogenesis and protection of the patient from the classical course of A-T [31, 32].

Located on chromosome 11q 22–23, the *ATM* gene comprises of 66 exons with a 9168 base pair coding sequence, and encodes a PI3K-related serine/threonine protein kinase (PIKK) that supports maintain genomic integrity. PIKK involves three structurally conserved domains, the N-spiral/solenoid/spiral, C-spiral/bridge/pincer, and the FAT (FRAP-ATM-TRRAP) [33]. *ATM* plays a main role in the repair of DNA double-strand breaks (DSB).

Together with spatial localization at the site of DSBs, *ATM* turns catalytically activated. The exact mechanism of this activation is not fully understood, but has been described to be associated with dissociation of ATM homodimers into active monomers, autophosphorylation of *ATM* at Ser1981, and acetylation of Lys3016 [34]. Once activated, *ATM* phosphorylates and activates other protein kinases. *ATM* also has effects on chromatin relaxation, which helps repair, through phosphorylation of KRAB-associated protein 1 (KAP1) and others [35].

The Ser1981 loop is not ordered in either the open or closed dimers, so it is unlikely that it controls the intrinsic activation state of the enzyme [36]. Therefore, ATM protein synthesized with the CNV could have residual ATM kinase activity despite the missing pincer domain or the missing Ser1981. Recently, Fiévet et al. highlighted the strength of KAP1 phosphorylation test for pathogenicity assessment and allowed the establishment of the A-T Atypical Score to predict atypical phenotype [37].

The majority of pathogenic variants are frameshift or nonsense alterations. These pathogenic variants cause total loss of ATM protein and explain the 75% of diagnosed A-T [18, 38]. Meanwhile, CNV is a rare *ATM* pathogenic variant type. Previous reports estimated that the large genomic alterations in *ATM* are detected in 1% to 12% of A-T patients [16–18].

To our knowledge, this is the first report of the atypical A-T with compound heterozygous SNV and CNV in *ATM* that showed normal level of AFP at diagnosis. The present report describes the two-year follow-up of gross motor function and laboratory data. We describe the two-year follow-up of clinical and laboratory data. We added this case to the 10 cases that were previously reported [18, 37, 39–42]. Martin-Rodriguez et al. reported the heterozygous pathogenic variants by a ∼90 kb genomic duplication spanning exons 17–61 and a missense variant (c.6899G > C) in exon 47 [39]. Huang et al. reported the heterozygous state in an A-T patient with a CNV in exon 63 and a nonsense variant (c.3174G > A) in exon 22 whose ataxia-age at onset was 48 months and serum levels of IgG, IgA, IgM, and Ig E were normal [18]. Podralska et al. identified a CNV of exons 62 and 63, combined with a nonsense variant c.5932G > T in exon 42 [40]. A general summary of the characteristics of the atypical A-T with compound heterozygous SNV and CNV in *ATM* are shown in Table 2.

**Table 2:** Features of 10 atypical A-T patients with compound heterozygous SNV and CNV in *ATM*

Patient	Sex	Age (year)	Phenotype (age, year)	Serum AFP	Pathogenic variants		Immunoglobulin levels (mg/dL)			References
					Allele 1	Allele 2	Ig G	Ig A	Ig M	
1	F	13	Ataxia (4) telangiectasia (1), cerebellar atrophy	325	Exon 22 c.3174G > A	Exon 63 Large genomic deletion	8800	1170	1070	Huang [18]
2	F	4	Start walking (1.5), movement abnormalities (1.5), normal brain MRI normal ophthalmologic exploration	214	Exon 47 c.6899G > C	Exon 17–61 g.(41245_49339)_ (137044_147250)dup	947	< 5	87	Martin-Rodriguez [39]
3	F	6	Ataxia (1.4), telangiectasia (3)	↑	Exon 62, 63 Large genomic deletion	Exon 42 c.5932G > T	↓	↓	NA	Podralska [40]
4	NA	NA	Ataxia < 8, typical phenotype	NA	Exon 18–33 c.(2638 + 1_2639–1)_ (5005 + 1_5006–1)dup	NA	NA	↓	NA	Fiévet [37]
5	NA	NA	Ataxia > 8, Loss of walking ability > 15, Oculomotor apraxia > 15	NA	Exon 17–61 c.(2466 + 1_2467–1)_ (8850 + 1_8851–1)dup	NA	NA	N	NA	Fiévet [37]
6	F	2	Ataxia (0.7), Absent presentation of telangiectasia, malignancy, respiratory disease, immunodeficiency	24	Exon 24 c.3576G > A; p.(Ser1135_Lys1192del58)	Exon NA c.(-31 + 1674_46) _(2405_2541)del (deletion)	NA	NA	NA	van Os [41]
7	NA	NA	NA	NA	Exon 07–17 deleted p.Arg111_Arg2191 > AsnfsX44	NA	NA	NA	NA	Micol [42]
8	NA	NA	NA	NA	Exon 08–15 deleted p.Glu166_Glu708 > AspfsX29	NA	NA	NA	NA	Micol [42]
9	NA	NA	NA	NA	Exon 59–61 deleted p.Val2758_Gly2891 > ValfsX46	NA	NA	NA	NA	Micol [42]
10	NA	NA	NA	NA	Exon 64–65 deleted p.Val2951_X3057 > SerfsX29	NA	NA	NA	NA	Micol [42]

*MRI* magnetic resonance imaging, *N* normal, *NA* not available, ↓ decreased (compared to age-related reference values), ↑ increased (compared to age-related reference values)

Our patient was confirmed as compound heterozygous for an *ATM* pathogenic SNV and a novel CNV by deletion of exons 24–40. Consistent with the previous reports, the compound heterozygosity by co-occurrence of a SNV and a CNV was correlated with late-onset and atypical phenotype.

We used a combination of NGS and Sanger sequencing technologies to cover the full coding regions of the listed genes. CNV was detected from VisCap, a CNV-detection and -visualization tool that compares the relative depth of read coverage across arbitrary sets of genome coordinates (e.g., exons) targeted in a set of DNA samples. VisCap provides graphical outputs to enable quality control and manual review with regard to exon-level data supporting and surrounding each CNV call [22].

CNVs are confirmed by technologies such as Comparative genomic hybridization (CGH), SNP array, Multiplex ligation-dependent probe amplification (MLPA), Polymerase Chain Reaction (PCR) or qPCR before they are reported. Our approach was one of the most cost-effective testing approaches, NGS with Sanger sequencing followed by the confirmation by SNP array.

SNP arrays are high-efficiency DNA microarrays that are a robust platform for simultaneously examining hundreds of thousands of SNPs and evaluating CNVs in a single experiment [43]. Recently, the cost of SNP arrays has decreased appreciably, driven by the very large sample sizes needed to perform genome-wide association studies. SNP arrays also allow allelotyping, generally small deletions/duplications need confirmation by an alternate method or are not detectable [44]. The drawback of SNP array includes inability to provide the exact breakpoint, hence, whether the deletion in the presenting report is in-frame or not needs further validating techniques.

Along with the microarray-based technologies, MLPA has also been the most reliable and effective methods for finding copy number variations. MLPA is a semiquantitative PCR-based technique that can discover deletions and duplications for up to 50 genetic loci in one assay [45]. The downside of MLPA involve inability to provide information in the context of the exact location of a duplicated sequence or its orientation, lack of sensitivity for regions not directly covered by the probe sets used, and false-positive results accountable for polymorphism-induced allele dropouts.

The majority of *ATM* pathogenic variants are truncating, generating highly unstable protein fragments in individuals with classic A-T. In such cases, ATM protein cannot be detected by western blotting and ATM kinase activity is not observed. Residual ATM protein is observable by western blot in individuals who possess reduced kinase activity and traditionally been referred to as “atypical” or “variant”. Moreover, certain missense variants, in-frame variants or leaky splice-site variants allow for the production of residual amounts of functioning ATM protein. Transcript analysis features the effect of the CNV and detects the nonsense-mediated decay.

Involvement of CNV in pathogenic variants may be missed in routine NGS-based diagnostics. Therefore, the clinical diagnosis of atypical A-T is difficult in patients whose genetic causes of disease involve CNV. Since there is little information available for the genotype and phenotype relationships in A-T variants due to co-occurrence of SNV and CNV, this case report reinforces the need to implement an integrated detection of heterogeneous pathogenic variants involving CNV if there is a doubt about the clinical diagnosis of atypical A-T.

The hallmarks of classical A-T are childhood-onset cerebellar ataxia, oculocutaneous telangiectasias, immunodeficiency with recurrent infections, pulmonary dysfunction, increased sensitivity to ionizing radiation, increased serum AFP levels and high risk of malignancies. Onset of symptoms usually occurs in early childhood by the age of 2 years with progressive cerebellar ataxia. Besides this classic phenotype, atypical phenotypes exist [33]. The disease course in such patients is characterized by atypical motor abnormalities [23]. Respiratory disease and immunodeficiency are not evident in atypical A-T, although these patients still have an increased cancer risk. Their lifespans are much longer compared to patients with the classic phenotype [23, 46].

Atypical A-T is frequently associated either with missense or leaky splice site variants that allow for some ATM protein with residual ATM kinase activity to be formed [32]. Podralska et al. reported a compound heterozygous with 2 CNVs in *ATM* that was related mixed phenotype involving onset of ataxia at 2 years old, decreased level of immunoglobulins and normal level of AFP [40]. Previous reports described mixed phenotypes encompassing typical and atypical clinical features in the atypical A-T patients with compound heterozygous SNV and CNV in *ATM* [18, 37, 39–42]. Consistent with the previous reports, the presenting report showed some typical features such as ataxia and ocular telangiectasia. Nevertheless, the patient showed normal level of AFP and immunology test results were borderline values at the diagnosis. Two-year follow-up data demonstrate that he could ambulate with walker and was medically stable although his gross motor and balance function declined, and the serum AFP level had risen over the course of two years. Apart from residual ATM kinase activity, other factors, such as modifying genes and environmental factors, such as comprehensive rehabilitation services are suggested to play a role in the mechanisms that lead to atypical phenotypes of A-T [39].

In conclusion, we reported a patient of A-T with delayed onset of ataxia and late increase of serum AFP level and identified the compound heterozygous *ATM* pathogenic variants involving a SNV of c.742C > T (p.Arg248Ter) and a novel CNV by the deletion of exons 24–40. Compound heterozygous *ATM* alterations including both SNV and CNV are rare and it may be associated with atypical phenotypes. Our findings will expand the genetic as well as the phenotypic spectrum of *ATM* pathogenic variants in A-T.

## Supplementary Information

**Additional file 1**. Annotation of the number of the exons.

**Additional file 2**. The Log ratio and the genomic position of the SNP around the breakpoint.

**Additional file 3**. Electrophoresis and Sanger sequencing of the RT-PCR product of an aberrant ATM mRNA.

## Data Availability

The Next Generation Sequencing datasets analyzed during the study are available in the NCBI Sequence Read Archive (SRA) with the bioproject accession number PRJNA750250. The copy number analysis including SNP array data generated in the study has been submitted to NCBI Gene Expression Omnibus (GEO) with the accession numbers GSE181612. Databases used in this study were NCBI BioProject (https://www.ncbi.nlm.nih.gov/bioproject/PRJNA750250), and NCBI GEO DataSets (https://www.ncbi.nlm.nih.gov/geo/query/acc.cgi?acc=GSE181612).

## Abbreviations

A-T: Ataxia-telangiectasia
AFP: Alpha fetoprotein
ATM: Ataxia telangiectasia mutated
CNV: Copy number variation
MRI: Magnetic resonance imaging
NGS: Next-generation sequencing
SNP: Single-nucleotide polymorphism
SNV: Single-nucleotide variant
