# Correction to: Compound heterozygous variants including a novel copy number variation in a child with atypical ataxia-telangiectasia: a case report

**DOI:** 10.1186/s12920-021-01086-8

**Published:** 2021-09-20

**Authors:** Hoo Young Lee, Dae-Hyun Jang, Jae-Won Kim, Dong-Woo Lee, Ja-Hyun Jang, Joungsu Joo

**Affiliations:** 1 TBI Rehabilitation Center, National Traffic Injury Rehabilitation Hospital, Gyeonggi-do, Republic of Korea; 2 Department of Rehabilitation Medicine, Seoul National University Hospital, Seoul, Republic of Korea; 3 Department of Medicine, Yonsei University College of Medicine, Seoul, Republic of Korea; 4 National Traffic Injury Rehabilitation Research Institute, National Traffic Injury Rehabilitation Hospital, Yangpyeong, Korea; 5 Department of Rehabilitation Medicine, Incheon St. Mary’s Hospital, College of Medicine, The Catholic University of Korea, 56, Dongsu-ro, Bupyeong-gu, Incheon, 21431 Republic of Korea; 6 Department of Laboratory Medicine and Genetics, Samsung Medical Center, Seoul, Korea; 7 EONE-DIAGNOMICS Genome Center, Incheon, Republic of Korea

## Correction to: BMC Med Genomics 14, 204 (2021) 10.1186/s12920-021-01053-3

Following publication of the original article [1], the authors reported an error in Fig. 2.

Figure 2 was replaced as the authors identified an error in part C that was introduced during the peer review process. This error may have affected the interpretation of the results, and therefore, the figure was replaced with the earlier correct version. The original article [1] has been corrected.

**Figure 2:**
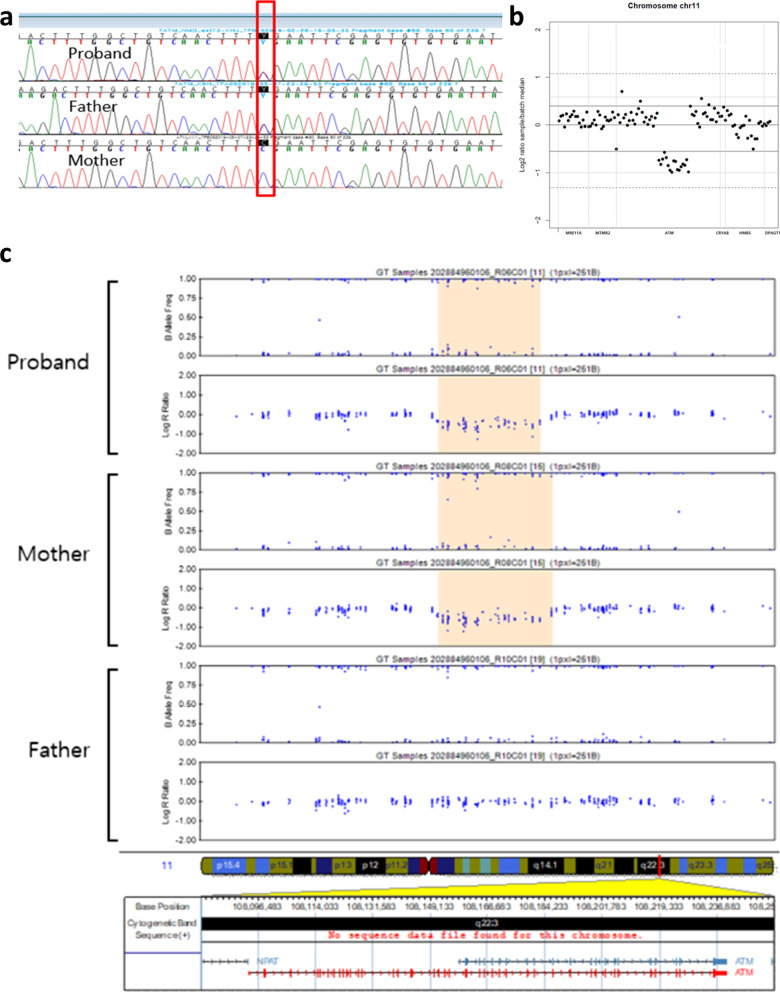
**a** Chromatograms of *ATM* sequence in the proband (top), the patient’s father (middle), and the patient’s mother (bottom) showing an SNV of c.742C > T (p.Arg248Ter) from the father. **b** Analysis of the next-generation sequencing data using VisCap. **c** SNP array analysis of the chromosome from the proband (top), the patient’s mother (middle), and the patient’s father (bottom) showing a novel CNV by the deletion of exons 24–40 from the mother. *SNV* single-nucleotide variation, *SNP* single-nucleotide polymorphism, *CNV* copy number variation
