# Supplementary material for: Compound heterozygous variants including a novel copy number variation in a child with atypical ataxia-telangiectasia: a case report
Source: BMC Med Genomics. 2021 Aug 17;14:204. doi: 10.1186/s12920-021-01053-3 (PMC8371864; doi:10.1186/s12920-021-01053-3)
Supplement: Supplementary file 1 — Additional file 1. Annotation of the number of the exons. [file 12920_2021_1053_MOESM1_ESM.docx]

**Supplementary material 1. Annotation of the number of the exons**

| **Exon** | **Start** | **End** | **Exon** | **Start** | **End** |
| --- | --- | --- | --- | --- | --- |
| 1 | 108,093,559 | 108,093,914 | 33 | 108,168,014 | 108,168,110 |
| 2 | 108,098,322 | 108,098,424 | 34 | 108,170,441 | 108,170,613 |
| 3 | 108,098,503 | 108,098,616 | 35 | 108,172,375 | 108,172,517 |
| 4 | 108,099,905 | 108,100,051 | 36 | 108,173,580 | 108,173,757 |
| 5 | 108,106,397 | 108,106,562 | 37 | 108,175,402 | 108,175,580 |
| 6 | 108,114,680 | 108,114,846 | 38 | 108,178,624 | 108,178,712 |
| 7 | 108,115,515 | 108,115,754 | 39 | 108,180,887 | 108,181,043 |
| 8 | 108,117,691 | 108,117,855 | 40 | 108,183,138 | 108,183,226 |
| 9 | 108,119,660 | 108,119,830 | 41 | 108,186,550 | 108,186,639 |
| 10 | 108,121,428 | 108,121,800 | 42 | 108,186,738 | 108,186,841 |
| 11 | 108,122,564 | 108,122,759 | 43 | 108,188,100 | 108,188,249 |
| 12 | 108,123,544 | 108,123,640 | 44 | 108,190,681 | 108,190,786 |
| 13 | 108,124,541 | 108,124,767 | 45 | 108,192,028 | 108,192,148 |
| 14 | 108,126,942 | 108,127,068 | 46 | 108,196,037 | 108,196,272 |
| 15 | 108,128,208 | 108,128,334 | 47 | 108,196,785 | 108,196,953 |
| 16 | 108,129,713 | 108,129,803 | 48 | 108,198,372 | 108,198,486 |
| 17 | 108,137,898 | 108,138,070 | 49 | 108,199,748 | 108,199,966 |
| 18 | 108,139,137 | 108,139,337 | 50 | 108,200,941 | 108,201,149 |
| 19 | 108,141,791 | 108,141,874 | 51 | 108,202,171 | 108,202,285 |
| 20 | 108,141,978 | 108,142,134 | 52 | 108,202,606 | 108,202,765 |
| 21 | 108,143,259 | 108,143,335 | 53 | 108,203,489 | 108,203,628 |
| 22 | 108,143,449 | 108,143,580 | 54 | 108,204,613 | 108,204,696 |
| 23 | 108,150,218 | 108,150,336 | 55 | 108,205,696 | 108,205,837 |
| 24 | 108,151,722 | 108,151,896 | 56 | 108,206,572 | 108,206,689 |
| 25 | 108,153,437 | 108,153,607 | 57 | 108,213,949 | 108,214,099 |
| 26 | 108,154,954 | 108,155,201 | 58 | 108,216,470 | 108,216,636 |
| 27 | 108,158,327 | 108,158,443 | 59 | 108,218,006 | 108,218,093 |
| 28 | 108,159,704 | 108,159,831 | 60 | 108,224,493 | 108,224,608 |
| 29 | 108,160,329 | 108,160,529 | 61 | 108,225,538 | 108,225,602 |
| 30 | 108,163,346 | 108,163,521 | 62 | 108,235,809 | 108,235,946 |
| 31 | 108,164,040 | 108,164,205 | 63 | 108,236,052 | 108,239,827 |
| 32 | 108,165,654 | 108,165,787 |  |  |  |
