# Supplementary material for: Compound heterozygous variants including a novel copy number variation in a child with atypical ataxia-telangiectasia: a case report
Source: BMC Med Genomics. 2021 Aug 17;14:204. doi: 10.1186/s12920-021-01053-3 (PMC8371864; doi:10.1186/s12920-021-01053-3)
Supplement: Supplementary file 2 — Additional file 2. The Log ratio and the genomic position of the SNP around the breakpoint. [file 12920_2021_1053_MOESM2_ESM.docx]

**Supplementary material 2. The Log ratio and the genomic position of the SNP around the breakpoint**

| exon | Location of SNPs | | Proband | | Mother | | Father | |
| --- | --- | --- | --- | --- | --- | --- | --- | --- |
|  | Chr | Position | LogR ratio | CNV | LogR ratio | CNV | LogR ratio | CNV |
| 23 | 11 | 108150302 | 0.020 | normal | -0.021 | normal | -0.111 | normal |
| 23 | 11 | 108150305 | 0.007 | normal | -0.031 | normal | 0.038 | normal |
| 23 | 11 | 108150314 | -0.127 | normal | -0.160 | normal | 0.043 | normal |
| 23 | 11 | 108150321 | -0.029 | normal | -0.129 | normal | -0.021 | normal |
| 24 | 11 | 108151766 | -0.389 | deleted | -0.444 | deleted | -0.100 | normal |
| 24 | 11 | 108151786 | -0.352 | deleted | -0.423 | deleted | 0.031 | normal |
| 24 | 11 | 108151843 | -0.420 | deleted | -0.354 | deleted | 0.083 | normal |
| 24 | 11 | 108151895 | -0.322 | deleted | -0.494 | deleted | 0.003 | normal |
| 25 | 11 | 108153485 | -0.420 | deleted | -0.510 | deleted | 0.063 | normal |
| 25 | 11 | 108153486 | -0.432 | deleted | -0.438 | deleted | 0.036 | normal |
| 25 | 11 | 108153490 | -0.677 | deleted | -0.719 | deleted | -0.298 | normal |
| 25 | 11 | 108153523 | -0.423 | deleted | -0.609 | deleted | -0.054 | normal |
| 25 | 11 | 108153528 | -0.730 | deleted | -0.820 | deleted | -0.214 | normal |
| 25 | 11 | 108153567 | -0.651 | deleted | -0.768 | deleted | 0.191 | normal |
| intron | 11 | 108154953 | NaN | - | -0.696 | deleted | 0.075 | normal |
| 26 | 11 | 108154961 | -0.556 | deleted | -0.610 | deleted | -0.118 | normal |
| 26 | 11 | 108154966 | -0.698 | deleted | -0.860 | deleted | -0.062 | normal |
| 26 | 11 | 108154970 | -0.813 | deleted | -0.835 | deleted | -0.341 | normal |
| 26 | 11 | 108154986 | -0.639 | deleted | -1.224 | deleted | -0.184 | normal |
| 26 | 11 | 108154986 | -0.436 | deleted | -0.786 | deleted | -0.051 | normal |
| 26 | 11 | 108155008 | -0.667 | deleted | -0.652 | deleted | 0.184 | normal |
| 26 | 11 | 108155043 | -0.623 | deleted | -0.207 | deleted | 0.164 | normal |
| 26 | 11 | 108155097 | -0.616 | deleted | -0.645 | deleted | -0.057 | normal |
| 26 | 11 | 108155097 | -0.399 | deleted | -0.573 | deleted | -0.012 | normal |
| 26 | 11 | 108155102 | -0.435 | deleted | -0.448 | deleted | -0.050 | normal |
| 26 | 11 | 108155138 | -0.552 | deleted | -0.584 | deleted | -0.211 | normal |
| 26 | 11 | 108155187 | -0.543 | deleted | -0.618 | deleted | -0.123 | normal |
| 26 | 11 | 108155201 | -0.410 | deleted | -0.531 | deleted | -0.021 | normal |
| intron | 11 | 108155205 | -0.560 | deleted | -0.538 | deleted | -0.062 | normal |
| intron | 11 | 108155240 | -0.469 | deleted | -0.506 | deleted | -0.038 | normal |
| intron | 11 | 108158325 | -0.514 | deleted | -0.395 | deleted | 0.124 | normal |
| 27 | 11 | 108158384 | -0.578 | deleted | -0.656 | deleted | 0.040 | normal |
| 27 | 11 | 108158399 | -0.586 | deleted | -0.680 | deleted | 0.015 | normal |
| intron | 11 | 108159703 | -0.754 | deleted | -0.869 | deleted | -0.318 | normal |
| intron | 11 | 108159703 | -0.734 | deleted | -0.862 | deleted | -0.175 | normal |
| 28 | 11 | 108159732 | -0.222 | deleted | -0.306 | deleted | -0.231 | normal |
| 28 | 11 | 108159733 | -0.766 | deleted | -0.921 | deleted | -0.256 | normal |
| 28 | 11 | 108159733 | -0.795 | deleted | -1.258 | deleted | -0.109 | normal |
| 28 | 11 | 108159742 | -0.535 | deleted | -0.570 | deleted | -0.134 | normal |
| 28 | 11 | 108159792 | -0.369 | deleted | -0.476 | deleted | -0.201 | normal |
| 28 | 11 | 108159820 | -0.302 | deleted | -0.537 | deleted | -0.153 | normal |
| intron | 11 | 108160327 | -0.744 | deleted | -0.786 | deleted | -0.067 | normal |
| 29 | 11 | 108160350 | -0.504 | deleted | -0.583 | deleted | -0.178 | normal |
| 29 | 11 | 108160371 | -0.890 | deleted | -1.034 | deleted | -0.486 | normal |
| 29 | 11 | 108160402 | -0.669 | deleted | -0.819 | deleted | -0.235 | normal |
| 29 | 11 | 108160416 | -0.722 | deleted | -0.637 | deleted | 0.007 | normal |
| 29 | 11 | 108160451 | -0.541 | deleted | -0.555 | deleted | 0.122 | normal |
| 29 | 11 | 108160454 | -0.626 | deleted | -0.641 | deleted | -0.031 | normal |
| 29 | 11 | 108160462 | -0.423 | deleted | -0.540 | deleted | 0.047 | normal |
| 29 | 11 | 108160464 | -0.578 | deleted | -0.589 | deleted | -0.053 | normal |
| 29 | 11 | 108160480 | -0.685 | deleted | -0.850 | deleted | -0.208 | normal |
| 29 | 11 | 108160486 | -0.615 | deleted | -0.735 | deleted | -0.126 | normal |
| 29 | 11 | 108160492 | -0.543 | deleted | -0.595 | deleted | 0.007 | normal |
| 29 | 11 | 108160516 | -0.593 | deleted | -0.680 | deleted | 0.113 | normal |
| 30 | 11 | 108163386 | -0.700 | deleted | -0.759 | deleted | -0.176 | normal |
| 30 | 11 | 108163483 | -0.436 | deleted | -0.779 | deleted | -0.173 | normal |
| 30 | 11 | 108163497 | -0.833 | deleted | -0.802 | deleted | -0.207 | normal |
| 31 | 11 | 108164051 | -1.296 | deleted | -0.930 | deleted | -0.245 | normal |
| 31 | 11 | 108164051 | -0.722 | deleted | -0.746 | deleted | -0.173 | normal |
| 31 | 11 | 108164092 | -0.616 | deleted | -0.605 | deleted | -0.300 | normal |
| 31 | 11 | 108164137 | -0.886 | deleted | -0.939 | deleted | -0.179 | normal |
| 31 | 11 | 108164150 | -0.789 | deleted | -0.890 | deleted | -0.279 | normal |
| 31 | 11 | 108164152 | -0.643 | deleted | -0.564 | deleted | -0.205 | normal |
| 31 | 11 | 108164163 | -0.786 | deleted | -0.753 | deleted | -0.284 | normal |
| intron | 11 | 108164206 | -0.947 | deleted | -0.710 | deleted | -0.134 | normal |
| 32 | 11 | 108165679 | -0.373 | deleted | -0.274 | deleted | -0.050 | normal |
| 32 | 11 | 108165729 | -0.328 | deleted | -0.273 | deleted | 0.000 | normal |
| 33 | 11 | 108168040 | -0.649 | deleted | -0.671 | deleted | -0.011 | normal |
| intron | 11 | 108169619 | -0.257 | deleted | -0.375 | deleted | -0.008 | normal |
| intron | 11 | 108170303 | -0.195 | deleted | -0.062 | deleted | -0.135 | normal |
| 34 | 11 | 108170449 | -0.331 | deleted | -0.465 | deleted | -0.036 | normal |
| 34 | 11 | 108170479 | -0.600 | deleted | -0.637 | deleted | 0.097 | normal |
| 34 | 11 | 108170524 | -0.701 | deleted | -0.812 | deleted | -0.012 | normal |
| 34 | 11 | 108170613 | -0.617 | deleted | -0.625 | deleted | -0.174 | normal |
| 35 | 11 | 108172383 | -0.569 | deleted | -0.660 | deleted | -0.098 | normal |
| 35 | 11 | 108172385 | -0.557 | deleted | -0.592 | deleted | -0.236 | normal |
| 35 | 11 | 108172397 | -0.544 | deleted | -0.587 | deleted | -0.045 | normal |
| 35 | 11 | 108172397 | -0.494 | deleted | -0.463 | deleted | 0.013 | normal |
| 35 | 11 | 108172425 | -0.318 | deleted | -0.438 | deleted | -0.036 | normal |
| 35 | 11 | 108172487 | -0.479 | deleted | -0.536 | deleted | -0.073 | normal |
| 35 | 11 | 108172506 | -0.399 | deleted | -0.574 | deleted | -0.039 | normal |
| intron | 11 | 108173575 | -0.694 | deleted | -0.733 | deleted | -0.159 | normal |
| 36 | 11 | 108173614 | -0.388 | deleted | -0.501 | deleted | -0.045 | normal |
| 36 | 11 | 108173656 | -0.456 | deleted | -0.432 | deleted | 0.098 | normal |
| 36 | 11 | 108173670 | -0.382 | deleted | -0.624 | deleted | -0.012 | normal |
| 36 | 11 | 108173674 | -0.360 | deleted | -0.440 | deleted | -0.055 | normal |
| intron | 11 | 108175400 | -0.470 | deleted | -0.603 | deleted | 0.110 | normal |
| 37 | 11 | 108175462 | -0.761 | deleted | -0.581 | deleted | -0.084 | normal |
| 37 | 11 | 108175463 | -0.463 | deleted | -0.657 | deleted | 0.041 | normal |
| 37 | 11 | 108175549 | -0.555 | deleted | -0.612 | deleted | -0.103 | normal |
| 37 | 11 | 108175556 | -0.520 | deleted | -0.660 | deleted | -0.105 | normal |
| 38 | 11 | 108178661 | -0.334 | deleted | -0.286 | deleted | -0.135 | normal |
| 38 | 11 | 108178712 | -0.355 | deleted | -0.296 | deleted | -0.090 | normal |
| intron | 11 | 108178926 | -0.529 | deleted | -0.635 | deleted | -0.167 | normal |
| 39 | 11 | 108180888 | -0.560 | deleted | -0.494 | deleted | -0.001 | normal |
| 39 | 11 | 108180915 | -0.678 | deleted | -0.696 | deleted | -0.192 | normal |
| 39 | 11 | 108180915 | -0.539 | deleted | -0.634 | deleted | -0.162 | normal |
| 39 | 11 | 108180982 | -0.535 | deleted | -0.526 | deleted | -0.037 | normal |
| 39 | 11 | 108181006 | -1.174 | deleted | -0.702 | deleted | -0.019 | normal |
| 39 | 11 | 108181014 | -0.731 | deleted | -0.766 | deleted | 0.113 | normal |
| 39 | 11 | 108181032 | -0.743 | deleted | -0.765 | deleted | -0.254 | normal |
| 39 | 11 | 108181033 | -0.532 | deleted | -0.677 | deleted | -0.342 | normal |
| 40 | 11 | 108183151 | -0.352 | deleted | -0.677 | deleted | -0.118 | normal |
| 40 | 11 | 108183190 | -0.538 | deleted | -0.743 | deleted | -0.078 | normal |
| 40 | 11 | 108183226 | -0.414 | deleted | -0.576 | deleted | -0.006 | normal |
| 41 | 11 | 108186590 | -0.123 | normal | -0.387 | ambiguous | -0.101 | normal |
| 41 | 11 | 108186610 | -0.146 | normal | -0.061 | ambiguous | -0.189 | normal |
| 41 | 11 | 108186631 | -0.139 | normal | -0.319 | ambiguous | 0.100 | normal |
| 41 | 11 | 108186638 | -0.178 | normal | -0.452 | ambiguous | -0.272 | normal |
| 41 | 11 | 108186639 | -0.311 | normal | -0.306 | ambiguous | -0.329 | normal |
| 42 | 11 | 108186742 | -0.212 | normal | -0.206 | ambiguous | -0.188 | normal |
| 42 | 11 | 108186796 | -0.045 | normal | -0.266 | ambiguous | -0.058 | normal |
| 42 | 11 | 108186818 | -0.069 | normal | -0.174 | normal | -0.100 | normal |
| 43 | 11 | 108188101 | -0.090 | normal | -0.197 | normal | -0.122 | normal |
| 43 | 11 | 108188128 | 0.110 | normal | -0.051 | normal | 0.086 | normal |
| 43 | 11 | 108188215 | -0.126 | normal | -0.133 | normal | -0.116 | normal |
| 43 | 11 | 108188227 | -0.003 | normal | -0.043 | normal | 0.048 | normal |
| 43 | 11 | 108188233 | 0.052 | normal | -0.205 | normal | -0.088 | normal |
