# Supplementary material for: Compound heterozygous variants including a novel copy number variation in a child with atypical ataxia-telangiectasia: a case report
Source: BMC Med Genomics. 2021 Aug 17;14:204. doi: 10.1186/s12920-021-01053-3 (PMC8371864; doi:10.1186/s12920-021-01053-3)
Supplement: Supplementary file 3 — Additional file 3. Electrophoresis and Sanger sequencing of the RT-PCR product of an aberrant ATM mRNA. [file 12920_2021_1053_MOESM3_ESM.docx]

**Supplementary material 3. a. Electrophoresis of the RT-PCR product shows a 347 bp band in the patient and mother. b. Sanger sequencing of the RT-PCR product shows an aberrant *ATM* mRNA with exons 24-40 completely deleted. Therefore, this pathogenic variant encompassed a novel CNV by in-frame deletion of exons 24–40**

| a. 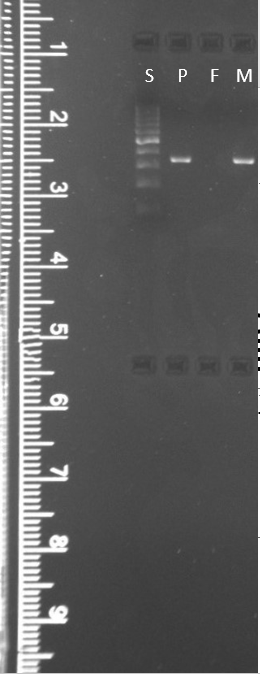 |
| --- |
| b. 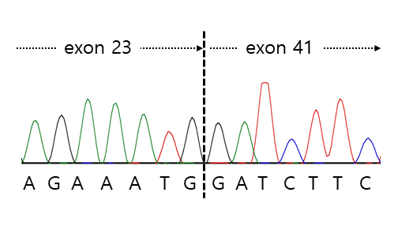 |

Abbreviations: RT-PCR; real-time polymerase chain reaction; bp; base pair; S: size marker; P: proband; F: father; M, mother.
